# Reference Hand-book of the Medical Sciences

**Published:** 1885-12

**Authors:** 


A REFERENCE HAND-BOOK OF THE MEDICAL SCIENCES. Being a complete and convenient work of reference for information upon topics belonging to the entire range of scientific and practical medicine, and consisting of a series of concise essays and brief paragraphs, arranged in the alphabetical order of the topics of which they treat, prepared by writers who are experts in their respective departments; illustrated by chromo lithographs and fine wood engravings. Edited by Albert H. Buck, M. D., New York city. Volume I. New York: William Wood & Company, 56 and 58 Lafayette Square. 1885.

For the medical man who desires varied information connected with his profession in a comparatively limited space, this work supplies the disideratum of a small, comprehensive library, as it is not confined to any department of knowledge, but covers the entire field of the medical sciences, including anatomy, physiology, surgery, obstetrics, gynecology, ophthalmology, practice of medicine, therapeutics, materia medica and botany, with many useful details of hygiene and important facts relating to the arts as connected with the medical profession.

That some defects must of necessity appear in an undertaking of such magnitude, in which there are so many contributors upon such a variety of subjects, is a reasonable expectation by the close observer.

The writers are for the most part well and favorably known by their previous productions, and, with the exception of eight from Canada, are residents of the different sections of the United States.

Out of ninety contributors to this first volume, there are only five who have not the title of M. D., and, as a consequence, the matters presented in it are to a very large extent purely of a professional nature.

A few points may be noted as departures from the recognized facts in some departments, such as the omission under the head of abdominal tumors, to refer to the large size of a pancreatic cyst removed by Bozeman, doubtless prior to the preparation of this article, not to speak of Senn's case, which has been more recently reported. The writer states that a pancreatic cyst "may, according to Bristowe, attain the size of an orange," whereas, that removed by Bozeman weighed twenty and a half pounds, and the one operated upon by Senn contained, according to his estimate, three quarts of fluid.

In the history of the discovery of anesthesia, there is no reference to the claim made and sustained in favor of Dr. Crawford W. Long, of Athens, Ga., having performed the first surgical operation under the influence of ether, and it is due to the truth that this fact should be recorded.

While describing, with some minuteness, the effects of the inhalation of chloroform, there is no allusion to the peculiar modification of the arterial blood from deficient oxygenation.

The article on asthma summarizes most of the ordinary means of treatment, without adverting to the efficacy of iodide of potash in bronchial asthma, or to the advantages of shower-baths in chronic cases of spasmodic asthma, which is a most important accompaniment of any internal measure of treatment.

Under the heading of Battey's Operation, it is said with justice, "to Dr. Battey is undoubtedly due the credit of demonstrating to the profession the practicability and value of an operation now all but universally admitted to an honored place in surgery." But the success of this operation has been overshadowed latterly by his performance of a series of ovariotomies without a single death, which could not have a place in the above article.

The otherwise commendable article on syphilitic diseases of the brain, by Dr. Eugene Foster, of Augusta, does not present such conclusive evidence of the dependence of epilepsy upon cerebral syphilis as to satisfy the requirements of the neurologist. When it is stated that the convulsive movements or phenomena may be the only prominent sign of cerebral syphilis, or they may be associated with other grave affections, we are left in the dark as to the cause and effect. It seems upon a still more dubious basis by the statement that "marked diminution of symptoms during a course of anti-syphilitic treatment is presumptive evidence of the syphilitic nature of the disease," as this is reasoning in a circle.

The well-elaborated paper upon anthrax has been reserved for notice in connection with the article having the designation, carbuncle, with a view to file an exception to the confusion of terms in styling malignant pustule, anthrax, when this latter term has been applied so generally to the inflammatory development known and here defined as carbuncle.

The importance of establishing clearly this distinction between two affections, which scarcely have any features of correspondence, would warrant a special article under the caption of malignant pustule, as distinguished from anthrax, in its appropriate place in the work. Either let us abandon the usual designation of carbuncle by the term *anthrax*, or cease to apply this appellation to malignant pustule, so that writers may be comprehended in using it. We may refer to an article on "the distinguishing features of malignant pustule and anthrax," in Gaillard's Journal for September, 1881, as setting forth distinctly the foundation for such a discrimination, by Littré, by Paulain et Garreau, commission des médicins de Chartres, and by Bouchust and Després in the Dictionaire Thérapeutique.

As there are so few short-comings to note, we prefer to commend this volume to the careful study of the medical men for their mature judgment upon its merits, with the assurance that this will prove to be the most useful and instructive of all medical publications made in the English language up to the present time. The illustrations, in the best style of artistic execution, which accompany the various contributions that require cuts for their better comprehension, constitute a notable feature of the work, while the clear, good-shaped type, in double columns, presents a most convenient page for the reader. The size and proportions of the volume make it very suitable as a hand-book for reference, and the quality of the paper, with the most substantial binding, does credit to the well-established fame of the publishers.

